# Pharmacogenomics Applied to Acute Leukemias: Identifying Clinically Relevant Genetic Variants

**DOI:** 10.3390/biomedicines13112581

**Published:** 2025-10-22

**Authors:** Flávia Melo Cunha de Pinho Pessoa, Isabelle Magalhães Farias, Beatriz Maria Dias Nogueira, Caio Bezerra Machado, Igor Valentim Barreto, Anna Karolyna da Costa Machado, Guilherme Passos de Morais, Leidivan Sousa da Cunha, Deivide de Sousa Oliveira, André Pontes Thé, Rodrigo Monteiro Ribeiro, Patrícia Maria Pontes Thé, Manoel Odorico de Moraes Filho, Maria Elisabete Amaral de Moraes, Caroline Aquino Moreira-Nunes

**Affiliations:** 1 Clinical Genetics Laboratory, Department of Medicine, Drug Research and Development Center (NPDM), Federal University of Ceará, Fortaleza 60430-275, CE, Brazil; flaviamelop@outlook.com (F.M.C.d.P.P.); isabellefariasbiomed@gmail.com (I.M.F.);; 2 Department of Hematology, Fortaleza General Hospital (HGF), Fortaleza 60150-160, CE, Brazil; 3 Clementino Fraga Group, Central Unity, Corporate Division, Fortaleza 60115-170, CE, Brazil; 4 Department of Pharmacy, Federal University of Ceará, Fortaleza 60020-181, CE, Brazil; 5 Clementino Fraga Group, Central Unity, Genomics and Molecular Biology Laboratory, Fortaleza 90619-900, CE, Brazil

**Keywords:** pharmacogenomics, acute myeloid leukemia, acute lymphoblastic leukemia, drug toxicity, pharmacogenomic variants

## Abstract

Acute leukemias are highly aggressive hematologic malignancies that demand intensive chemotherapy regimens. However, drug toxicity remains a major barrier to treatment success and patient survival. In this context, pharmacogenomics offers a promising strategy by identifying single-nucleotide variants (SNVs) that influence drug metabolism, efficacy, and toxicity, ultimately impacting treatment outcomes. This study analyzed data from the ClinPGx/PharmGKB database to identify clinically annotated variants related to chemotherapy response in Acute Myeloid Leukemia (AML) and Acute Lymphoblastic Leukemia (ALL). A total of 24 variants were curated for AML and 57 for ALL. Among these, nonsynonymous variants were most frequent in ALL (31.6%), while synonymous variants predominated in AML (33.3%). Although traditionally considered neutral, synonymous and intronic variants may influence gene expression through regulatory or splicing mechanisms. The analysis revealed clinically significant variants associated with chemotherapy response, particularly in the *ABCB1* gene, observed in 12.5% of AML and 10.5% of ALL cases. Several variants, particularly *TPMT*, *NUDT15*, *ABCC1*, *SLC28A3*, and *RARG*, were associated with severe adverse effects such as myelotoxicity, mucositis, cardiotoxicity, and hepatotoxicity. This study reinforces the importance of genetic variants in modulating the therapeutic response and toxicity to chemotherapy drugs in acute leukemias. Analysis of ClinPGx/PharmGKB data emphasizes *ABCB1* as a potential resistance marker and supports pre-treatment genotyping of genes like *TPMT* and *NUDT15* to prevent severe toxicities. Future advances should include the expansion of pharmacogenetic studies in underrepresented populations and the clinical validation of new markers in prospective trials, aiming to consolidate precision medicine as a routine part of the therapeutic management of acute leukemias.

## 1. Introduction

Acute leukemias represent a group of hematological malignancies of rapid progression and high lethality, which are characterized by the uncontrolled proliferation of immature hematopoietic cells [1,2]. Despite advances in diagnosis and clinical management, conventional chemotherapy remains the main therapeutic modality for these diseases. However, the chemotherapy regimens used are often associated with severe side effects, with toxicity being one of the main obstacles in treatment. Many patients progress to severe complications resulting from chemotherapy, which can compromise the continuity of treatment and, in more extreme cases, lead to death [3,4,5].

Faced with this challenging scenario, there is a global effort in the search for more effective and safer therapies, such as target-directed drugs, which promise a more personalized and less aggressive approach [6]. However, while these treatments are still in the consolidation phase for many forms of acute leukemia, variability in response to conventional chemotherapy remains a critical factor to consider.

Within this context, pharmacogenomics plays a key role in investigating how genetic variations affect drug response. Through the analysis of single-nucleotide variants (SNVs) it is possible to predict drug effectiveness and make dose adjustments in a personalized way. Genetic variants can be classified as synonymous, which do not alter the amino acid sequence of the encoded protein, or non-synonymous, which include missense mutations, nonsense mutations which introduce a premature stop codon, and frameshift mutations. Among these, non-synonymous variants, particularly nonsense and frameshift mutations, are generally more deleterious, as they tend to disrupt protein structure and function more severely [7,8].

As genomic technology advances and sequencing costs decreases, the integration of pharmacogenomics into clinical practice becomes increasingly feasible, enabling more precise and individualized care for patients [9,10,11]. Despite its potential, pharmacogenomic studies in acute leukemia are still scarce, especially in diverse populations, which limits the clinical application of this knowledge.

Therefore, this study aimed to compile all variants described in the ClinPGx/PharmGKB database for acute myeloid leukemia (AML) and acute lymphoblastic leukemia (ALL), identifying the main effects of the presence of each variant in relation to the efficacy of treatment with chemotherapy and the adverse effects resulting from drug toxicity.

## 2. Methodology

### Search Strategy

Secondary data were obtained from the ClinPGx/PharmGKB, a public database that curate information on how genetic variation affects drug response. This resource integrates data from the scientific literature and clinical studies to support pharmacogenomic research. [12]. In this study, the ClinPGx/PharmGKB was used to identify all genetic variants previously reported to influence chemotherapy response in acute leukemias, without restriction by publication period. Variants related to acute myeloid or lymphoblastic leukemia were retrieved from the “Clinical Annotations” section, which compiles evidence of their clinical relevance, and further explored through the “Variant Annotations” section to review supporting studies. For each variant, data were collected on genomic location, functional impact, and associations with drug efficacy and toxicity, excluding those related solely to metabolism or dosage adjustment. Population allele frequencies were sourced from the 1000 Genomes Project (Phase 3), using the five major superpopulations: African (AFR), Admixed American (AMR), East Asian (EAS), European (EUR), and South Asian (SAS) [13]. The data were curated manually and with the aid of Microsoft Excel spreadsheets, where the variants were initially organized by gene, mutation type, population, and described clinical association. Functional annotations were complemented using literature references indicated by the database itself. After filtering, the variants were reorganized considering the associated gene, type of drug involved, and reported clinical impact.

The level of evidence (LOE) for gene variant–drug associations is a classification made by ClinPGx/PharmGKB that ranges from 1A to 4, based on the strength of available data. Level 1A represents the highest level of evidence, describing variant–drug combinations with specific guidelines in clinical practice guidelines or Food and Drug Administration (FDA) drug labels, requiring at least one additional supporting scientific publication. Level 1B also reflects strong evidence but does not include specific recommendations, requiring two independent publications.

Levels 2A and 2B include associations with moderate evidence, differentiated by the presence (2A) or absence (2B) of variants in ClinPGx/PharmGKB’s Very Important Pharmacogenes (VIPs), both requiring two supporting publications. Level 3 includes associations with limited evidence, which may be based on single studies or preliminary data. Notably, Level 4 is assigned when evidence is insufficient or contradicts the association, resulting in a negative score. This hierarchical classification system enables systematic evaluation of the reliability of gene variant-drug response associations.

## 3. Results

Initial screening of clinical notes available in the ClinPGx/PharmGKB database resulted in a total of 25 records of drug response-associated variants related to acute myeloid leukemia (AML) and 106 records associated with acute lymphoblastic leukemia (ALL). After applying the eligibility criteria, such as the presence of specific information on genetic variants (SNVs) and their association with therapeutic response or toxicity to drugs used in the treatment of acute leukemias, 24 annotations were selected for AML (Table 1) and 57 for ALL (Table 2).

In total, all 24 variants identified in AML related to chemotherapy had a level of evidence (LOE) of 3. These variants are distributed in 17 genes, and the genes with the highest frequency of variants were *ABCB1* (12.5%) and *RRM2* (12.5%). Among the findings, most of the variants found are synonymous (33.33%) and non-synonymous (20.83%). They have a variety of associations with several drugs, with cytarabine (79.16%) and idarubicin (54.16%) being the drugs that were most present in correlation with the variants.

**Figure 1:**
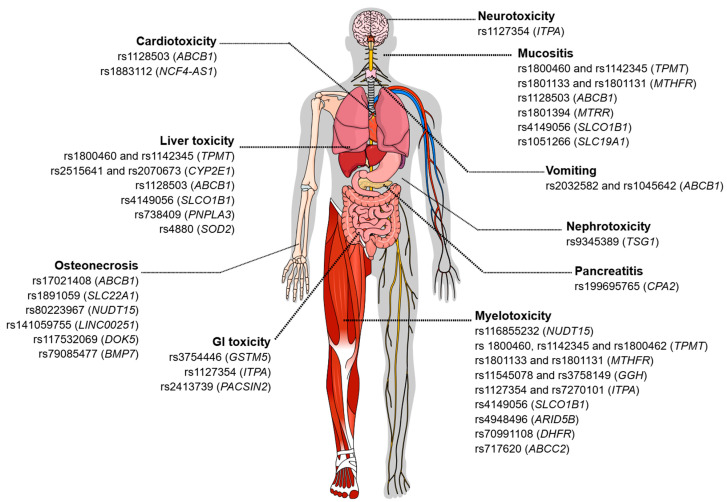
Genetic variants associated with toxicity related to the treatment of acute leukemias. The figure illustrates different types of chemotherapy-induced toxicities and the genetic variants described in the literature that are associated with each adverse manifestation. Among the toxicities represented are neurotoxicity, cardiotoxicity, hepatotoxicity, pancreatitis, gastrointestinal toxicity, nephrotoxicity, myelotoxicity, mucositis, vomiting, and osteonecrosis. For each type of toxicity, single-nucleotide polymorphisms (SNPs) and the corresponding genes potentially involved in individual susceptibility to these adverse events are indicated. Figure created in the Mind the Graph platform (www.mindthegraph.com (accessed on 3 September 2025)).

Depending on the genotype (heterozygous or homozygous mutated), an association was observed with the increase or reduction in the intensity of these adverse effects, with 75% of these effects being associated with the efficacy of the drugs and 50% associated with toxicity. The associations regarding the efficacy of chemotherapy drugs are still controversial. About 50% of the reported associations show that the presence of certain variants is related to a higher probability of overall survival (OS), event-free survival (EFS), complete remission (CR), and response to treatment. However, the presence of other specific variants confers a worse response. The main symptomatic characteristics observed regarding toxicity were vomiting, toxic liver disease, and cardiotoxicity (Figure 1).

In general, altered alleles are associated with a worse response/reaction to chemotherapy drugs. However, in AML, some variants showed positive effects related to the presence of the alternative allele or the homozygous genotype for this allele. This is the case of the rs80143932 and rs2306744 variants, located in the *DCK* gene, whose altered alleles are associated with a greater response to treatment using cytarabine and idarubicin. In addition, the rs1042919 (*RRM1*) variant, in turn, demonstrates that the presence of alleles—reference and altered—in heterozygosis, may be associated with a decrease in the response to treatment using cladribine and cytarabine in children.

In contrast to most cases, the absence of the rs1130609 (*RRM2*) and rs1561876 (*STIM1*) variants, i.e., the presence of only the reference alleles, is associated with decreased therapeutic response in children. Similarly, the rs11231825 (*SLC22A12*) variant demonstrates that the homozygous genotype of the reference allele may be related to a higher probability of fever occurrence.

Overall, the 57 variants in ALL are listed in 37 genes that are associated with the response to chemotherapy, of which 4 were level 1A, 1 level 2A, 49 level 3 and 3 level 4. The most frequently listed genes were *ABCB1* (10.5%), *GGH* (7%), *NUDT15, TPMT,* and *SLCO1B1* (5.2% each). The types of variants that occurred most frequently were variants and non-synonyms (31.6%) and intronics (28%). The drugs with the greatest relations with variants were methotrexate (80.7%), followed by asparaginase (21%), vincristine (19.3%) and mercaptopurine (15.7%), mostly being associated with a poorer response and/or increased toxicity.

The presence of variants, whether homozygous or heterozygous, has been associated with the modulation of the intensity of adverse effects, which may result in an increase or reduction in these events. Approximately 38.6% of these associations refer to therapeutic efficacy, in which the presence of at least one allele altered with the variant is associated with minimal residual disease (MRD) detection after induction, decreased event-free survival (EFS) and response to treatment, and increased likelihood of relapse and resistance. Meanwhile, about 84.2% of variants are related to drug toxicity, with the main reported toxic effects being myelosuppression, leukopenia, neutropenia/febrile neutropenia, thrombocytopenia, mucositis, toxic liver disease, osteonecrosis, and gastrointestinal toxicity (Figure 1).

Among the four variants classified with LOE 1A, one occurs in the *NUDT15* gene (non-synonymous) and the other 3 were located in the *TPMT* gene (2 non-synonymous and 1 exonic/splicing). Notably, none of these variants were synonymous, which is consistent with their well-established functional impact on protein activity and, consequently, on drug metabolism. All were associated with toxicity related to chemotherapy agents mercaptopurine, thioguanine and methotrexate. The presence of mutated alleles generally increased the likelihood of myelosuppression, severe pancytopenia, and drug-related toxicity in patients.

The non-synonymous rs1801133 variant that occurs in the *MTHFR* gene has evidence level 2A and showed associations with the use of methotrexate. According to studies, the presence of the altered allele could indicate a greater probability of relapses and a higher risk of drug toxicity, mucositis, thrombocytopenia, leukopenia, neutropenia, and myelosuppression.

**Figure 2:**
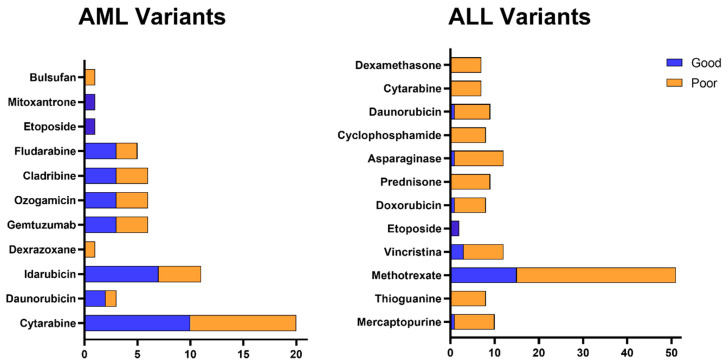
Distribution of genetic variants associated with the response to drugs used in the treatment of acute leukemias. Association between genetic variants and response to the main chemotherapeutic agents used in the treatment of AML and ALL. The figure shows the prognosis associated with the genetic variants previously described in the tables, classified as related to good response (blue) or poor response (orange) for each therapeutic agent. Drugs depicted include alkylating agents, anthracyclines, topoisomerase II inhibitors, antimetabolites, enzymes, and conjugated antibodies.

Figure 2 shows that, among the genetic variants described in the previous tables, the relationship with unfavorable outcomes predominates, either due to reduced therapeutic efficacy or increased risk of toxicity. Although variants related to good response are also present, they are less frequently distributed. It is also observed that certain drugs, such as cytarabine, daunorubicin, L-asparaginase, and methotrexate, concentrate a greater number of variants associated with a negative prognosis, while others, such as mercaptopurine and cyclophosphamide, have a more balanced profile between beneficial and adverse effects. Cytarabine stands out in particular, which not only brings together several variants, but is also related to genetic alterations with a relevant clinical impact on the therapeutic response.

Some variants of the *ABCB1* gene are common between AML and ALL, such as rs1045642 and rs1128503. The rs1045642 in AML patients treated with cytarabine is associated with higher chances of complete remission and 3-year EFS, in addition to also increasing the probability of vomiting and increased liver enzymes [15,16,17,22]. In ALL patients treated with vincristine, methotrexate and etoposide, the absence of this same variant was associated with a higher probability of developing leukopenia, neutropenia, mucositis, hepatotoxicity, anemia, and thrombocytopenia [45,47,53,54,55,113,114].

The presence of SNV rs1128503 in AML patients treated with cytarabine is associated with lower overall survival (OS) and higher probability of cardiotoxicity. While in methotrexate-treated ALL patients, the presence of the variant is related to a higher risk of drug-induced liver injury and increased severity of mucositis.

In general, the presence of genetic variants is commonly related to a worse response or greater toxicity to chemotherapy treatment; however, in some cases the opposite is observed. The absence of the rs1127354 (*ITPA*), rs408626, and rs442767 (*MSH3*) variants is associated with a higher risk of leukopenia. The presence of rs1051266 (*SLC19A1*) variant in homozygosis is related to greater severity in mucositis, whereas its absence is associated with a higher probability of toxic liver disease, neutropenia, pancreatitis, vomiting, and myelosuppression.

## 4. Discussion

The investigation of genetic variants associated with therapeutic response in acute leukemia has been shown to be of great relevance in view of the high interindividual variability in the response to chemotherapy drugs and the significant frequency of serious adverse events related to drug toxicity. Understanding these changes allows the personalization of the treatment, contributing to the choice of safer and more effective therapeutic regimens, in addition to minimizing potentially lethal adverse reactions.

The survival rate in AML (32.9%) is generally lower than in ALL (92% for children and 40–60% for adults). This is due to the fact that AML is more prevalent in adults and the elderly, who generally tolerate intensive chemotherapy poorly. It is a disease with lower sensitivity to conventional chemotherapy, with fewer target-directed therapeutic options, and with more common and rapid relapses. ALL, on the other hand, is more prevalent in children, who usually respond and tolerate chemotherapy protocols well, unlike adult patients, who have a slightly worse prognosis. Still, in recent years ALL has evolved in recent decades with standardized protocols and greater use of targeted therapies, such as blinatumomab and inotuzumab [3,119,120,121,122]. Even so, there are fewer studies on the pharmacogenetics of AML and the influence of genetic variants on the response to chemotherapy drugs than on ALL.

Regarding ALL, most studies address the pediatric population, but over the years there has been an increase in the absolute number of cases in adult patients, as reported by Yi et al. (2020) who state that between 1990 and 2017 there was an increase of around 31% in cases [123]. Given their poorer prognosis, further studies should explore how variants affect this patient group, with the aim of reducing severe toxicities and improving clinical outcomes.

In this study, it was possible to observe a predominance of non-synonymous variants (missense) in both types of leukemias, especially in ALL. These variants are generally associated with a greater functional potential, as they cause amino acid substitutions that can directly affect the structure and function of proteins involved in the metabolization, transport or action of chemotherapy drugs. Some studies have already observed that the presence of this type of variant influences the response to drugs used in the treatment of acute leukemia in different ways and may alter the effectiveness of the treatment or increase the risk of severe toxicities [122,124].

On the other hand, the high percentage of synonymous variants observed in AML should not be underestimated. Although these variants do not directly alter the protein sequence, there is growing evidence that they can influence drug response through altering mRNA stability, translation efficiency, and especially splicing. In cancer, synonymous variants in genes such as *ABCB1* (*MDR1*) can modulate the expression of efflux proteins, influencing the response to chemotherapy drugs [125].

In this research, we identified *ABCB1* as the gene with the highest frequency of single-nucleotide variants (SNVs) associated with chemotherapy response in acute leukemias, found in 12.5% of AML and 10.5% of ALL cases. *ABCB1* (*MDR1*) encodes P-glycoprotein (P-gp), an ATP-dependent efflux pump that removes cytotoxic drugs from cells, such as anthracyclines, vincristine, and etoposide. Functional studies and pharmacogenetic analyses demonstrate that *ABCB1* polymorphisms affect P-gp expression and activity, and are significantly correlated with treatment failure, refractory disease, relapse, reduced event-free and overall survival, as well as increased hematologic toxicity [126,127,128]. Furthermore, a meta-analyses indicated that variant alleles of *ABCB1* influence drug accumulation in blast cells, improving remission rates but also elevating toxicity risk [129].

Although hematologic cancers typically exhibit relatively low P-gp levels, post-treatment genetic instability, and clonal selection in refractory or relapsed patients enhance *ABCB1* activation and P-gp overexpression. Beyond drug efflux, P-gp appears to support leukemic blast survival through mechanisms independent of chemotherapy expulsion—such as regulation of apoptotic pathways [130,131,132]. These data collectively reinforce the critical role of *ABCB1* SNVs in mediating chemoresistance and underscore the utility of integrating *ABCB1* genotyping into precision therapy for acute leukemia.

The identification of high-risk *ABCB1* variants raises the question of how to clinically overcome P-gp mediated resistance. One promising strategy involves co-administration of P-gp inhibitors to restore intracellular chemotherapeutic concentrations. While third-generation inhibitors like tariquidar have faced toxicity challenges in trials, repurposing established drugs such as verapamil and cyclosporine A has shown promise in preclinical models, and newer agents like elacridar represent a translational frontier for patients with resistance profiles [133,134].

A comprehensive understanding of pharmacogenomics in chemoresistance must also account for key genes with the highest levels of clinical evidence, such as *TPMT* and *NUDT15*, which are critical for thiopurine drug metabolism. Both genes carry a Level 1A evidence rating from the Clinical Pharmacogenetics Implementation Consortium (CPIC), underscoring their well-validated role in clinical practice. Thiopurine S-methyltransferase (*TPMT*) catalyzes the S-methylation of thiopurines, inactivating them and preventing excessive formation of cytotoxic thioguanine nucleotides (TGNs). Patients with loss-of-function alleles (e.g., *TPMT2*, *3A*, *3C*) accumulate TGNs, leading to high risk of severe myelosuppression [135].

Similarly, *NUDT15* provides a critical detoxification pathway by hydrolyzing the active metabolite deoxythioguanosine triphosphate (dTGTP), preventing its misincorporation into DNA. Loss-of-function *NUDT15* variants (e.g., rs116855232) result in dTGTP accumulation, causing DNA damage and leukopenia. For patients carrying these risk variants, pharmacological interventions are already clinically validated; for instance, allopurinol supplementation mitigates thiopurine-induced hematologic toxicity through “allopurinol-guided dosing,” allowing safe administration of therapeutic doses [136,137]. Pre-emptive genotyping for *TPMT* and *NUDT15*, enabling dose reductions or therapy switching, exemplifies the successful translation of pharmacogenetics into clinical practice.

It is noteworthy that none of the LOE 1A variants were synonymous, reinforcing that non-synonymous and splicing variants, which directly alter protein function, demonstrate stronger clinical associations [11,138]. However, the potential impact of synonymous and noncoding variants should not be disregarded. While de current pharmacogenomic landscape is dominated by functionally disruptive variants, emerging evidence highlights the role of synonymous and intronic variants in regulating gene expression through mechanisms such as altered mRNA stability, translation efficiency, splicing modulation, and epitranscriptomic modifications like N6-adenosine methylation (m6A) [139,140,141,142]. For example, intronic variants in ALL, though noncoding, can alter splicing or regulatory elements, influencing drug response [143]. Therefore, future studies should include functional annotation of synonymous and intronic polymorphisms to fully elucidate the genetic landscape of chemoresistance [125,144,145].

The adverse effects observed in the treatment of acute leukemia directly reflect the pharmacological nature of the chemotherapy agents, whose targets are tissues of high turnover and metabolic pathways that are fundamental for cell proliferation. The presence of genetic variants associated with the response to chemotherapy drugs can influence the frequency and intensity of these effects and can generate such toxicity that it can lead to the patient’s death even before the treatment protocol is completed [63,146,147].

Polymorphisms in genes such as *TPMT* (e.g., 3A, 3C) and *NUDT15* (e.g., rs116855232) are strongly associated with severe myelotoxicity in patients with acute leukemia treated with thiopurines, such as mercaptopurine and thioguanine. The presence of these variants, even in heterozygosis, can increase the risk of severe myelosuppression by up to nine times, requiring significant dose reductions and may culminate in fatal outcomes in the absence of adequate therapeutic adjustment [36].

Similarly, variants in genes such as *SLC28A3*, *ABCC1*, *RARG*, *UGT1A6*, and *ABCB4*, involved in anthracycline transport and metabolism, have been implicated in the predisposition to cardiotoxicity in pediatric and adult patients, compromising treatment continuity and may negatively affect late survival [73,148,149,150]. These recent findings corroborate the importance of pre-therapy genetic evaluation to mitigate severe toxicities, individualizing the approach, and improving clinical outcomes in ALL and AML patients.

While transcriptomic analyses provide valuable insights into gene expression states, a comprehensive genomic profiling strategy must also incorporate the systematic SNV identification to fully predict treatment outcomes and toxicity risks. Gene expression levels are transient and influenced by various factors, whereas germline and somatic SNVs represent stable, patient-specific markers that directly alter protein function, drug metabolism, and transport. Relying solely on expression data may miss critical pharmacogenetic variants in genes such as *DPYD*, *TPMT*, *NUDT15*, or *ABCB1*, which may not correlate with mRNA abundance but profoundly impact drug efficacy and safety. Therefore, integrating SNV profiling with RNA-sequencing or whole-genome sequencing in clinical workflows enables more robust personalized risk assessment, combining correlative expression signatures with causative genetic factors to refine therapeutic decisions [11,151].

Overall, most reported variants have a LOE of 3, indicating limited supporting data from single studies, case reports, or in vitro assays [12]. This highlights the need for further research to validate these associations and elucidate their clinical impact. While the use of the ClinPGx/PharmGKB database was instrumental in curating clinically relevant pharmacogenetic associations, our study’s limitations reflect those of the source resource, including potential population bias due to underrepresentation of non-European cohorts, underrepresentation of rare variants, and the predominance of LOE 3 associations. Therefore, our results should be interpreted as a comprehensive yet evolving landscape, underscoring the imperative for further validation in diverse cohorts and functional studies to enable safer, more personalized chemotherapeutic interventions.

## 5. Conclusions

This study reinforces the critical role of genetic variants in modulating therapeutic response and toxicity to chemotherapeutic agents in acute leukemias. Our comprehensive survey of the ClinPGx/PharmGKB database revealed a diverse landscape of pharmacogenomic variants in AML and ALL, with the *ABCB1* gene exhibiting the highest frequency in both subtypes, highlighting its central role in mediating resistance. More importantly, the numerous associations identified between specific variants and severe adverse effects highlight the tangible clinical value of pre-treatment genotyping. The implementation of point-of-care testing for high-evidence genes, such as *TPMT* and *NUDT15*, represents a readily actionable strategy to proactively guide initial dosing, prevent life-threatening toxicities like myelosuppression, and improve a patient’s quality of life during treatment.

To fully realize the potential of precision oncology in acute leukemias, future efforts must extend beyond current knowledge. It is essential to expand pharmacogenetic studies, particularly in underrepresented populations, to ensure the equity and global applicability of genotyping panels. Furthermore, the clinical utility of a broader set of variants, including those involved in resistance mechanisms like *ABCB1* and other promising markers with preliminary evidence, must be rigorously validated in prospective clinical trials. In these trials, patients would be randomized to receive either standard therapy or a genotype-guided protocol, with endpoints focusing on the reduction in severe adverse events, improved dose intensity, and enhanced overall survival. Success of such initiatives would pave the way for the systematic integration of comprehensive pharmacogenomics into standard treatment protocols, ultimately shifting the paradigm from reactive toxicity management to proactive, personalized therapy that maximizes efficacy and safety for all patients.

## Figures and Tables

**Table 1:** Acute Myeloid Leukemia Drug Response-Associated Variants Annotations.

Gene	dbSNP150	Ref	Alt	Annotation	LOE *	Phenotype Categories	Variant Frequencies * (%)	Drugs	Efficacy Association	Toxicity Association	References
*ABCB1*	rs2032582	A	C	nsSNV	3	Efficacy, Toxicity	AFR: 97.96	cytarabine, daunorubicin, dexrazoxane, idarubicin	Genotype CC is associated with decreased OS, increased likelihood of CR and increased likelihood of 3-year EFS.	Genotype CC is associated with increased likelihood of vomiting.	[14,15,16,17]
							AMR: 57.20				
							EAS: 46.83				
							EUR: 57.26				
							SAS: 35.79				
*CD33*	rs35112940	G	A	nsSNV	3	Efficacy	AFR: 2.50	gemtuzumab ozogamicin	Genotype GG is associated with increased OS.	-	[18]
							AMR: 10.95				
							EAS: 0.00				
							EUR: 21.17				
							SAS: 3.58				
*NOS3*	rs1799983	T	G	nsSNV	3	Efficacy	AFR: 92.97	daunorubicin	Genotype GG is associated with increased OS.	-	[19]
							AMR: 78.53				
							EAS: 87.00				
							EUR: 65.61				
							SAS: 83.23				
*RRM2*	rs1130609	T	G	nsSNV	3	Efficacy	AFR: 95.16	cladribine, cytarabine	Allele T is associated with decreased response to treatment in children.	-	[20]
							AMR: 54.76				
							EAS: 33.93				
							EUR: 73.26				
							SAS: 55.93				
*SLCO1B1*	rs4149056	T	C	nsSNV	3	Toxicity	AFR: 1.36	cytarabine, fludarabine, gemtuzumab ozogamicin, idarubicin	-	Allele C is associated with increased likelihood of toxic liver disease.	[21]
							AMR: 13.40				
							EAS: 12.30				
							EUR: 16.10				
							SAS: 4.29				
*ABCB1*	rs1045642	A	G	sSNV	3	Efficacy, Toxicity	AFR: 85.02	cytarabine	Genotype GG is associated with increased likelihood of CR and 3-year EFS. Allele A is associated with increased OS.	Genotype GG is associated with increased likelihood of vomiting and elevated liver enzymes.	[15,16,17,22]
							AMR: 57.20				
							EAS: 60.22				
							EUR: 48.21				
							SAS: 42.54				
*ABCB1*	rs1128503	A	G	sSNV	3	Efficacy, Toxicity	AFR: 86.38	cytarabine	Genotype GG is associated with decreased OS. Allele A is associated with increased OS.	Genotype GG is associated with increased likelihood of cardiotoxicity.	[14,17,22]
							AMR: 59.65				
							EAS: 37.30				
							EUR: 58.45				
							SAS: 41.31				
*CYP2E1*	rs2515641	C	T	sSNV	3	Toxicity	AFR: 66.11	cytarabine, fludarabine, gemtuzumab, ozogamicin, idarubicin	-	Allele T is associated with decreased likelihood of toxic liver disease.	[21]
							AMR: 17.87				
							EAS: 20.54				
							EUR: 12.82				
							SAS: 16.77				
*NT5C3A*	rs3750117	A	G	sSNV	3	Efficacy	AFR: 81.39	cytarabine, idarubicin	Genotype GG is associated with increased risk of induction failure.	-	[23]
							AMR: 71.76				
							EAS: 46.13				
							EUR: 70.87				
							SAS: 60.12				
*RRM2*	rs5030743	C	G	sSNV	3	Efficacy	AFR: 9.91	cladribine, cytarabine	Allele G is associated with decreased response to treatment in children.	-	[20]
							AMR: 0.58				
							EAS: 0.00				
							EUR: 0.00				
							SAS: 0.00				
*SLC22A12*	rs11231825	T	C	sSNV	3	Toxicity	AFR: 9.53	cytarabine, fludarabine, gemtuzumab ozogamicin, idarubicin	-	Genotype TT is associated with increased likelihood of fever.	[21]
							AMR: 43.23				
							EAS: 22.12				
							EUR: 70.58				
							SAS: 58.69				
*SLCO1B1*	rs2291075	C	T	sSNV	3	Efficacy	AFR: 55.98	cytarabine, daunorubicin, etoposide, mitoxantrone	Allele T is associated with increased EFS and OS.	-	[24]
							AMR: 33.14				
							EAS: 51.09				
							EUR: 39.66				
							SAS: 20.14				
*SULT2B1*	rs2302948	C	T	sSNV	3	Toxicity	AFR: 19.14	cytarabine, fludarabine, gemtuzumab ozogamicin, idarubicin	-	Allele T is associated with decreased likelihood of fever.	[21]
							AMR: 16.71				
							EAS: 8.83				
							EUR: 24.06				
							SAS: 14.11				
*CDA*	rs532545	C	T	uSNV	3	Efficacy, Toxicity	AFR: 5.90	cytarabine	Genotype TT is associated with decreased 5-year survival and increased risk of death.	Genotype TT is associated with increased cytotoxicity.	[22,25,26]
							AMR: 30.84				
							EAS: 11.71				
							EUR: 30.32				
							SAS: 22.19				
*CYP2E1*	rs2070673	A	T	uSNV	3	Toxicity	AFR: 23.98	cytarabine, fludarabine, gemtuzumab ozogamicin, idarubicin	-	Allele A is associated with decreased likelihood of toxic liver disease.	[21]
							AMR: 66.71				
							EAS: 54.07				
							EUR: 81.61				
							SAS: 61.86				
*DCK*	rs80143932	C	G	uSNV	3	Efficacy	AFR: 1.59	cytarabine, idarubicin	Allele G is associated with increased response to treatment.	-	[27]
							AMR: 5.19				
							EAS: 15.77				
							EUR: 1.09				
							SAS: 5.32				
*DCK*	rs2306744	C	T	5′UTR SNV	3	Efficacy	AFR: 1.66	cytarabine, idarubicin	Allele T is associated with increased response to treatment.	-	[27]
							AMR: 5.19				
							EAS: 15.77				
							EUR: 1.09				
							SAS: 5.21				
*STIM1*	rs1561876	G	A	3′UTR SNV	3	Efficacy	AFR: 29.50	cladribine, cytarabine	Genotype GG is associated with decreased response to treatment in children.	-	[20]
							AMR: 77.67				
							EAS: 76.69				
							EUR: 88.37				
							SAS: 90.49				
*RRM1*	rs1042919	A	T	3′UTR SNV	3	Efficacy	AFR: 79.65	cladribine, cytarabine	Genotype AT is associated with decreased response to treatment in children.	-	[20]
							AMR: 82.56				
							EAS: 75.99				
							EUR: 92.94				
							SAS: 92.64				
*GSTM5*	rs3754446	A	C	igSNV	3	Toxicity	AFR: 1.74	bulsufan	-	Allele C is associated with increased likelihood of GI toxicity in children.	[28,29]
							AMR: 46.97				
							EAS: 68.85				
							EUR: 33.70				
							SAS: 37.01				
*RRM2B*	rs1265138	A	G	igSNV	3	Efficacy	AFR: 13.39	cladribine, cytarabine	Genotype AA is associated with increased response to treatment in children.	-	[20]
							AMR: 8.36				
							EAS: 31.45				
							EUR: 4.57				
							SAS: 25.56				
*NCF4-AS1*	rs1883112	G	A	ncRNA_iSNV	3	Efficacy, Toxicity	AFR: 12.93	idarubicin	Allele A is associated with increased response to idarubicin.	Allele A is associated with increased risk of cardiotoxicity.	[30]
							AMR: 51.59				
							EAS: 66.57				
							EUR: 42.25				
							SAS: 45.81				
*RAC2*	rs13058338	T	A	iSNV	3	Efficacy, Toxicity	AFR: 8.55	idarubicin	Allele A is associated with increased response to idarubicin.	Allele A is associated with decreased risk of drug toxicity.	[30]
							AMR: 24.78				
							EAS: 6.94				
							EUR: 26.44				
							SAS: 18.40				
*STIM1*	rs2898950	A	C	iSNV	3	Efficacy	AFR: 47.96	cladribine, cytarabine	Allele C is associated with increased response to treatment in children	-	[20]
							AMR: 90.63				
							EAS: 96.03				
							EUR: 91.95				
							SAS: 93.35				

Ref: reference allele; Alt: altered allele; LOE: level of evidence; nsSNV: nonsynonymous single-nucleotide variant; sSNV: synonymous single-nucleotide variant; igSNV: intergenic single-nucleotide variant; iSNV: intronic single-nucleotide variant; uSNV: upstream single-nucleotide variant; ncRNA_iSNV: noncoding RNA intronic single-nucleotide variant; 5′UTR SNV: 5′ untranslated region single-nucleotide variant; 3′UTR SNV: 3′ untranslated region single-nucleotide variant; AFR: African; AMR: admixed American; EAS: East Asian; EUR: European; SAS: South Asian; EFS: event-free survival; OS: overall survival; CR: complete remission; GI: gastrointestinal; -: information not reported. * 1000 Genomes Project frequency of the altered allele.

**Table 2:** Acute Lymphoblastic Leukemia Drug Response-Associated Variants Annotations.

Gene	dbSNP150	Ref	Alt	Annotation	LOE *	Phenotype Categories	Variant Frequencies * (%)	Drugs	Efficacy Association	Toxicity Association	References
*NUDT15*	rs116855232	C	T	nsSNV	1A	Toxicity	AFR: 0.08	mercaptopurine	-	Allele T is associated with increased likelihood of leukopenia. Genotype CT is associated with increased likelihood of severe myelosuppression, anemia, neutropenia, and thrombocytopenia.	[31,32,33,34,35,36]
							AMR: 4.47				
							EAS: 9.52				
							EUR: 0.20				
							SAS: 6.95				
*TPMT*	rs1800460	C	T	nsSNV	1A	Toxicity	AFR: 0.30	thioguanine	-	TPMT*3A is associated with increased severity of hepatic veno-occlusive disease, myelosuppression, elevated liver enzymes, drug toxicity, infection, stomatitis, neutropenia, and thrombocytopenia. *TPMT*3C is associated with increased likelihood of leukopenia, neutropenia, and severe pancytopenia.	[37,38,39,40,41]
							AMR: 4.03				
							EAS: 0.00				
							EUR: 2.78				
							SAS: 0.41				
*TPMT*	rs1142345	T	C	nsSNV	1A	Toxicity	AFR: 6.66	mercaptopurine, tioguanine			
							AMR: 5.76				
							EAS: 2.18				
							EUR: 2.88				
							SAS: 1.74				
*TPMT*	rs1800462	C	G	eSNV	1A	Toxicity	AFR: 0.08	mercaptopurine, tioguanine, methotrexate	-	*TPMT*2 is associated with increased risk of drug toxicity, neutropenia and thrombocytopenia, and decreased likelihood of febrile neutropenia.	[40,42,43,44]
							AMR: 0.58				
							EAS: 0.00				
							EUR: 0.60				
							SAS: 0.00				
*MTHFR*	rs1801133	G	A	nsSNV	2A	Efficacy, Toxicity	AFR: 9.00	methotrexate	Genotype GG is associated with increased EFS. Genotype AA is associated with increased likelihood of relapse.	Allele A is associated with increased likelihood of drug toxicity, treatment interruption, mucositis, thrombocytopenia, leukopenia, neutropenia, and myelosuppression.	[45,46,47,48,49,50,51,52]
							AMR: 47.41				
							EAS: 29.56				
							EUR: 36.48				
							SAS: 11.86				
*ABCB1*	rs1045642	A	G	sSNV	3	Toxicity	AFR: 85.02	vincristine, methotrexate, etoposide	-	Genotype AA is associated with increased likelihood of leukopenia, neutropenia or mucositis, toxic liver disease, anemia, thrombocytopenia, and toxicity. Allele A is associated with decreased likelihood of neurotoxicity syndromes.	[45,47,53,54,55,56]
							AMR: 57.20				
							EAS: 60.22				
							EUR: 48.21				
							SAS: 42.54				
*ABCB1*	rs1128503	A	G	sSNV	3	Toxicity	AFR: 86.38	methotrexate	-	Allele G is associated with increased likelihood of drug-induced liver injury and severe mucositis.	[47,57,58,59]
							AMR: 59.65				
							EAS: 37.30				
							EUR: 58.45				
							SAS: 41.31				
*ABCB1*	rs2229109	C	T	nsSNV	3	Efficacy	AFR: 0.30	doxorubicin, methotrexate, prednisolone, vincristine	Genotype CT is associated with increased resistance.	-	[56]
							AMR: 2.45				
							EAS: 0.00				
							EUR: 3.28				
							SAS: 0.92				
*FOLH1*	rs61886492	G	A	nsSNV	3	Toxicity	AFR: 3.33	mercaptopurine, methotrexate	-	Allele A is associated with increased risk of drug toxicity.	[60]
							AMR: 2.74				
							EAS: 0.10				
							EUR: 5.07				
							SAS: 3.07				
*GGH*	rs11545078	G	A	nsSNV	3	Toxicity	AFR: 5.60	methotrexate	-	Allele A is associated with increased likelihood of thrombocytopenia.	[61]
							AMR: 4.03				
							EAS: 8.73				
							EUR: 9.24				
							SAS: 14.83				
*GGH*	rs11545077	C	T	nsSNV	3	Efficacy	AFR: 10.51	methotrexate	Allele C is associated with increased response to treatment.	-	[62]
							AMR: 20.46				
							EAS: 21.83				
							EUR: 25.05				
							SAS: 25.77				
*GSTP1*	rs1695	A	G	nsSNV	3	Toxicity	AFR: 48.03	mercaptopurine, methotrexate	-	Genotype GG is associated with increased likelihood of drug toxicity.	[63]
							AMR: 47.55				
							EAS: 17.86				
							EUR: 33.10				
							SAS: 29.45				
*ITPA*	rs1127354	C	A	nsSNV	3	Efficacy, Toxicity	AFR: 4.46	methotrexate	Genotype CC is associated with increased EFS.	Allele A is associated with increased risk of GI toxicity, neurotoxicity syndromes, neutropenia, and severe myelosuppression. Genotype AC is associated with increased severity of febrile neutropenia. Genotype CC is associated with increased risk of leukopenia.	[32,64,65,66,67,68]
							AMR: 4.18				
							EAS: 16.87				
							EUR: 7.06				
							SAS: 12.17				
*MTHFD1*	rs2236225	G	A	nsSNV	3	Efficacy, Toxicity	AFR: 15.81	methotrexate	Allele A is associated with decreased likelihood of EFS.	Allele A is associated with decreased risk of toxic liver disease.	[69,70]
							AMR: 54.47				
							EAS: 19.84				
							EUR: 42.94				
							SAS: 50.41				
*MTRR*	rs1801394	A	G	nsSNV	3	Toxicity	AFR: 24.58	methotrexate	-	Allele G is associated with decreased likelihood of toxic liver disease and increased likelihood of mucositis.	[71,72]
							AMR: 28.10				
							EAS: 26.29				
							EUR: 52.29				
							SAS: 52.45				
*PNPLA3*	rs738409	C	G	nsSNV	3	Toxicity	AFR: 11.80	asparaginase, cyclophosphamide, daunorubicin, prednisolone, vincristine	-	Allele G is associated with increased likelihood of elevated liver enzymes and toxic liver disease.	[73,74,75]
							AMR: 48.41				
							EAS: 35.02				
							EUR: 22.56				
							SAS: 24.64				
*SHMT1*	rs1979277	G	A	nsSNV	3	Toxicity	AFR: 33.13	methotrexate	-	Genotype AG is associated with decreased likelihood of toxic liver disease.	[72]
							AMR: 26.95				
							EAS: 6.05				
							EUR: 30.82				
							SAS: 14.83				
*SLCO1B1*	rs4149056	T	C	nsSNV	3	Efficacy, Toxicity	AFR: 1.36	methotrexate	Genotypes Allele C is associated with decreased likelihood of relapse.	Allele C is associated with increased likelihood of mucositis, thrombocytopenia, and neutropenia. Genotype TT is associated with increased severity of infection.	[59,71,73,76,77]
							AMR: 13.40				
							EAS: 12.30				
							EUR: 16.10				
							SAS: 4.29				
*SOD2*	rs4880	A	G	nsSNV	3	Toxicity	AFR: 42.36	asparaginase	-	Genotype GG is associated with increased concentrations of bilirubin and risk of toxic liver disease.	[78,79]
							AMR: 58.36				
							EAS: 12.50				
							EUR: 46.62				
							SAS: 50.82				
*CCND1*	rs9344	G	A	eSNV	3	Efficacy, Toxicity	AFR: 18.76	methotrexate	Genotype AA is associated with decreased EFS.	Genotype AA is associated with decreased severity of drug toxicity.	[80,81]
							AMR: 34.87				
							EAS: 57.14				
							EUR: 49.70				
							SAS: 51.64				
*CPA2*	rs199695765	C	T	eSNV	3	Toxicity	NR	asparaginase	-	Allele T is associated with increased risk of pancreatitis.	[82]
*BMP7*	rs79085477	C	T	igSNV	3	Toxicity	AFR: 5.82	Protocol *	-	Allele T is associated with increased risk of osteonecrosis.	[83]
							AMR: 0.72				
							EAS: 6.85				
							EUR: 0.80				
							SAS: 3.48				
*DOK5*	rs117532069	G	A	igSNV	3	Toxicity	AFR: 0.23	Protocol *	-	Allele A is associated with increased risk of osteonecrosis.	[83]
							AMR: 1.15				
							EAS: 0.00				
							EUR: 1.79				
							SAS: 0.00				
*FPGS*	rs1544105	C	T	igSNV	3	Efficacy, Toxicity	AFR: 61.95	methotrexate	Genotype TT is associated with increased response to treatment.	Genotype TT is associated with decreased likelihood of GI toxicity.	[29,84]
							AMR: 48.13				
							EAS: 69.05				
							EUR: 39.66				
							SAS: 40.08				
*GRIA1*	rs4958381	T	C	igSNV	3	Toxicity	AFR: 11.65	asparaginase	-	Allele T is associated with increased risk of allergy.	[85]
							AMR: 8.79				
							EAS: 12.40				
							EUR: 4.08				
							SAS: 13.80				
*LINC00251*	rs141059755	A	G	igSNV	3	Toxicity	AFR: 0.00	Protocol *	-	Allele G is associated with increased risk of osteonecrosis.	[83]
							AMR: 0.72				
							EAS: 0.00				
							EUR: 0.10				
							SAS: 0.00				
*NUDT15*	rs80223967	A	G	igSNV	3	Toxicity	AFR: 4.99	Protocol **	-	Allele G is associated with increased risk of osteonecrosis.	[83]
							AMR: 3.46				
							EAS: 0.00				
							EUR: 6.16				
							SAS: 1.64				
*SLC22A1*	rs1891059	G	A	igSNV	3	Toxicity	AFR: 2.57	Protocol **	-	Allele A is associated with increased risk of osteonecrosis.	[83]
							AMR: 3.31				
							EAS: 0.00				
							EUR: 5.86				
							SAS: 1.64				
*ABCB1*	rs17021408	T	C	igSNV	3	Toxicity	AFR: 6.13	Protocol **	-	Allele C is associated with increased risk of osteonecrosis.	[83]
							AMR: 3.89				
							EAS: 0.00				
							EUR: 5.96				
							SAS: 1.64				
*IL6R*	rs4888024	A	G	igSNV	3	Efficacy	AFR: 30.56	methotrexate	Allele G is associated with end-of-induction MRD.	-	[86]
							AMR: 58.21				
							EAS: 86.31				
							EUR: 38.87				
							SAS: 55.11				
*ABCB1*	rs4728709	G	A	iSNV	3	Toxicity	AFR: 40.70	vincristine	-	Allele A is associated with decreased risk of neurotoxicity syndromes.	[87]
							AMR: 13.98				
							EAS: 13.00				
							EUR: 6.06				
							SAS: 6.03				
*ABCC2*	rs3740065	A	G	iSNV	3	Toxicity	AFR: 21.71	methotrexate		Allele G is associated with increased risk of toxicity.	[88]
							AMR: 9.94				
							EAS: 30.95				
							EUR: 11.83				
							SAS: 22.49				
*ABCC4*	rs7317112	A	G	iSNV	3	Toxicity	AFR: 67.02	methotrexate	-	Genotype AA is associated with increased risk of mucositis.	[77,89,90]
							AMR: 29.83				
							EAS: 36.61				
							EUR: 25.94				
							SAS: 24.64				
*ARID5B*	rs4948496	T	C	iSNV	3	Toxicity	AFR: 70.50	methotrexate	-	Allele C is associated with increased risk of leukopenia.	[91]
							AMR: 59.08				
							EAS: 63.49				
							EUR: 49.70				
							SAS: 61.25				
*CAT*	rs10836235	C	T	iSNV	3	Toxicity	AFR: 9.23	anthracyclines and related substances	-	Genotype CC is associated with increased risk of cardiac damage.	[92]
							AMR: 10.52				
							EAS: 30.36				
							EUR: 9.44				
							SAS: 3.27				
*MSH3*	rs442767	G	T	iSNV	3	Toxicity	AFR: 3.18	methotrexate	Genotype GG is associated with decreased EFS	Allele G is associated with increased risk of fatigue and leukopenia.	[93,94,95]
							AMR: 39.91				
							EAS: 58.53				
							EUR: 31.91				
							SAS: 2 2.80				
*DHFR*	rs70991108	-	TGGCGCGTCCCGCCCAGGT	Fs-ins	3	Toxicity	NR	methotrexate	-	Allele del is associated with increased risk of drug-induced liver injury; Altered allele is associated with increased severity of leukopenia and thrombocytopenia.	[51,96,97]
*MSH3*	rs408626	T	C	iSNV	3	Efficacy, Toxicity	AFR: 56.20	methotrexate	Genotype CC is associated with decreased EFS and OS.	Genotype TT is associated with increased risk of leukopenia.	[93,95]
							AMR: 51.01				
							EAS: 60.22				
							EUR: 45.03				
							SAS: 29.96				
*DROSHA*	rs639174	C	T	iSNV	3	Toxicity	AFR: 56.88	cyclophosphamide, cytarabine, daunorubicin, mercaptopurine, methotrexate, prednisone, vincristine	-	Allele T is associated with increased risk of drug toxicity.	[98]
							AMR: 33.14				
							EAS: 69.44				
							EUR: 27.44				
							SAS: 33.64				
*ITPA*	rs7270101	A	C	iSNV	3	Toxicity	AFR: 7.11	mercaptopurine, methotrexate	-	Allele C is associated with increased likelihood of thrombocytopenia, leukopenia, and neutropenia.	[37,68]
							AMR: 8.21				
							EAS: 0.00				
							EUR: 12.92				
							SAS: 1.53				
*NFATC2*	rs6021191	A	T	iSNV	3	Toxicity	AFR: 17.10	asparaginase	-	Allele T is associated with increased risk of hypersensitivity.	[99]
							AMR: 3.75				
							EAS: 10.42				
							EUR: 0.00				
							SAS: 3.58				
*PACSIN2*	rs2413739	C	T	iSNV	3	Efficacy, Toxicity	AFR: 48.49	mercaptopurine, methotrexate	Allele T is associated with increased risk of relapse.	Genotype TT is associated with increased risk of adverse events and GI toxicity.	[100,101,102,103]
							AMR: 35.01				
							EAS: 7.24				
							EUR: 43.74				
							SAS: 42.84				
*PYGL*	rs7142143	T	C	iSNV	3	Efficacy	AFR: 11.20	asparaginase, dexamethasone, methotrexate	Allele C is associated with increased risk of relapse.	-	[104]
							AMR: 0.14				
							EAS: 0.00				
							EUR: 0.20				
							SAS: 0.00				
*SLC19A1*	rs2838958	G	A	iSNV	3	Efficacy	AFR: 17.40	methotrexate	Genotype AA is associated with decreased response to treatment.	-	[74]
							AMR: 42.36				
							EAS: 44.44				
							EUR: 52.88				
							SAS: 59.92				
*SLCO1B1*	rs4149081	G	A	iSNV	3	Toxicity	AFR: 18.84	methotrexate	-	Allele G is associated with increased risk of GI toxicity.	[105,106]
							AMR: 17.15				
							EAS: 45.34				
							EUR: 18.99				
							SAS: 8.18				
*SLCO1B1*	rs11045879	T	C	iSNV	3	Toxicity	AFR: 18.91	mercaptopurine; methotrexate	-	Allele T is associated with increased risk of mucositis and GI toxicity.	[73,101,105,106]
							AMR: 17.29				
							EAS: 45.34				
							EUR: 18.99				
							SAS: 8.18				
*TSG1*	rs9345389	A	G	ncRNA_iSNV	3	Efficacy, Toxicity	AFR: 12.78	methotrexate	Allele G is associated with end-of-induction MRD in children.	Allele G is associated with increased risk of nephrotoxicity.	[86,90]
							AMR: 15.56				
							EAS: 22.22				
							EUR: 0.70				
							SAS: 6.54				
*ABCC3*	rs9895420	T	A	uSNV	3	Efficacy, Toxicity	AFR: 21.03	methotrexate	Allele A is associated with increased risk of relapse in the CNS and decreased EFS.	Allele A is associated with decreased likelihood of thrombocytopenia.	[107]
							AMR: 6.20				
							EAS: 8.43				
							EUR: 12.23				
							SAS: 11.25				
*GGH*	rs3758149	G	A	uSNV	3	Efficacy, Toxicity	AFR: 16.7	methotrexate	Allele A is associated with increased response to treatment.	Genotype AA is associated with increased likelihood of anemia.	[62,108]
							AMR: 22.77				
							EAS: 21.92				
							EUR: 27.83				
							SAS: 28.63				
*ABCC2*	rs717620	C	T	5′UTR SNV	3	Toxicity	AFR: 3.10	methotrexate	-	Allele T is associated with increased likelihood of drug toxicity and leukopenia.	[77,91,109]
							AMR: 16.57				
							EAS: 21.73				
							EUR: 20.68				
							SAS: 9.51				
*DHFR*	rs1105525	C	T	5′UTR SNV	3	Efficacy	AFR: 3.10	methotrexate	Allele T is associated with decreased EFS.	-	[110]
							AMR: 11.53				
							EAS: 7.44				
							EUR: 16.30				
							SAS: 26.18				
*GGH*	rs11545076	A	C	5′UTR SNV	3	Efficacy	AFR: 16.72	methotrexate	Allele C is associated with increased response to treatment.	-	[62]
							AMR: 22.77				
							EAS: 21.92				
							EUR: 27.93				
							SAS: 28.63				
*TYMS*	rs11280056	TTAAAG	-	ifdel	3	Toxicity	NR	methotrexate	-	Reference allele is associated with increased likelihood of GI toxicity and neutropenia.	[97,111]
*NUDT15*	rs746071566	GGAGTC	-	ifdel	3	Toxicity	NR	mercaptopurine	-	*NUDT15*1/4 (assigned as intermediate metabolizer phenotype) is associated with increased risk of leukopenia and neutropenia.	[44,112]
*ABCB1*	rs1045642	A	G	sSNV	4	Efficacy	AFR: 85.02	vincristine, methotrexate, etoposide	Allele A is associated with decreased response to treatment.	-	[45,47,53,54,55,113,114]
							AMR: 57.20				
							EAS: 60.22				
							EUR: 48.21				
							SAS: 42.54				
*MTHFR*	rs1801131	T	G	nsSNV	4	Efficacy, Toxicity	AFR: 15.13	methotrexate	Genotype TT is associated with increased EFS and with decreased response to treatment.	Allele G is associated with increased severity of neutropenia and risk of mucositis, decreased risk of skin disorder, and myelosuppression.	[51,52,72,97,115,116]
							AMR: 15.13				
							EAS: 21.92				
							EUR: 31.31				
							SAS: 41.72				
*SLC19A1*	rs1051266	T	C	nsSNV	4	Efficacy, Toxicity	AFR: 32.68	methotrexate	Genotype TT is associated with increased likelihood of staying in remission.	Genotype CC is associated with increased severity of mucositis; Genotype TT is associated with increased likelihood of toxic liver disease, mucositis, neutropenia, pancreatitis, vomiting and myelosuppression.	[35,51,55,56,117,118]
							AMR: 58.21				
							EAS: 47.42				
							EUR: 54.87				
							SAS: 59.41				

Ref: reference allele; Alt: altered allele; LOE: level of evidence; nsSNV: nonsynonymous single-nucleotide variant; sSNV: synonymous single-nucleotide variant; igSNV: intergenic single-nucleotide variant; iSNV: intronic single-nucleotide variant; uSNV: upstream single-nucleotide variant; ncRNA_iSNV: noncoding RNA intronic single-nucleotide variant; eSNV: exonic single-nucleotide variant; ifdel: in-frame deletion; Fs-ins: frameshift insertion; 5′UTR SNV: 5′ untranslated region single-nucleotide variant; AFR: African; AMR: Admixed American; EAS: East Asian; EUR: European; SAS: South Asian; NR: Not Reported; EFS: event-free survival; OS: overall survival; MRD: minimal residual disease; CNS: central nervous system; GI: gastrointestinal; -: information not reported. * 1000 Genomes frequency of the altered allele. ** Protocol: Cyclophosphamide, cytarabine, daunorubicin, dexamethasone, doxorubicin, methotrexate, pegaspargase, prednisone, thioguanine and vincristine. These drugs are used in different combinations and treatment phases in several well-established protocols such as BFM (ALL-BFM), COG (e.g., AALL0331, AALL1131), and St. Jude Total Therapy.

## Data Availability

The data presented in this study are publicly available. Information on pharmacogenetic variants was obtained from the PharmGKB database (https://www.clinpgx.org/), and variant frequency data were obtained from the 1000 Genomes Project (https://www.coriell.org/). All data sources are properly cited in the manuscript.
